# Dual equipoise shared decision making: definitions for decision and behaviour support interventions

**DOI:** 10.1186/1748-5908-4-75

**Published:** 2009-11-18

**Authors:** Glyn Elwyn, Dominick Frosch, Stephen Rollnick

**Affiliations:** 1 Clinical Epidemiology Interdisciplinary Research Group, Department of Primary Care and Public Health, School of Medicine, Cardiff University, Heath Park, CF14 4YS, UK; 2 Department of Health Services Research, Palo Alto Medical Foundation Research Institute, 795 El Camino Real, Palo Alto, CA 94301 USA

## Abstract

**Background:**

There is increasing interest in interventions that can support patients who face difficult decisions and individuals who need to modify their behaviour to achieve better outcomes. Evidence for effectiveness is used to categorise patients care. Effective care is where evidence of benefit outweighs harm: patients should always receive this type of care, where indicated. Preference-sensitive care describes a situation where the evidence for the superiority of one treatment over another is either not available or does not allow differentiation; in this situation, there are two or more valid approaches, and the best choice depends on how individuals value the risks and benefits of treatments.

**Discussion:**

Preference-sensitive decisions are defined by equipoise: situations where options need to be deliberated. Moreover, where both healthcare professionals and patients agree that equipoise exists, situations may be regarded as having 'dual equipoise'. Such conditions are ideal for shared decision making. However, there are many situations in medicine where dual equipoise does not exist, where health professionals hold the view that scientific evidence for benefit strongly outweighs harm. This is often the case where people suffer from chronic conditions, and where behaviour change is recommended to improve outcomes. However, some patients, are either ambivalent or find it difficult to sustain optimal behaviours, *i.e*., patients will be in varying degrees of equipoise. Therefore, situations where dual equipoise exists (or not) help to clarify the definitions of two classes of support, namely, decision and behaviour change support interventions. Decision support interventions help people think about choices they face; they describe where and why choice exists, in short, conditions of dual equipoise; they provide information about options, including, where reasonable, the option of taking no action. These interventions help people to deliberate, independently or in collaboration with others, about options by considering relevant attributes; they support people to forecast how they might feel about short, intermediate, and long-term outcomes that have relevant consequences, in ways that help the process of constructing preferences and eventual decision making appropriate to their individual situation. Whereas, behavioural support interventions describe, justify, and recommend actions that, over time, lead to predictable outcomes over short, intermediate, and long-term timeframes, and that have relevant and important consequences for those who are considering behaviour change.

**Summary:**

Decision and behaviour support interventions have divergent aims, different relationships to equipoise, and form two classes of interventions.

## Background

The interest in creating interventions that help patients to make decisions about treatments or tests, or to help people considering immunisations, screening tests, and other choices in healthcare, has led to substantial debate in how these tools should be developed, designed, and implemented [1]. Although interventions to support decision making have been developed in decision science for over 50 years, it is only over the last decade or so that we have seen a significant interest in interventions specifically designed for patients. These interventions are known by a number of different names (shared decision-making programs, decision aids, decision support tools or technologies), a nomenclature indicative of a field that has developed rapidly since the late 1990s [2]. The increasing number of such interventions underlines the fact that medicine is undergoing a significant shift in how the roles of physician and patient are defined. At the heart of this shift is the recognition that decisions in medicine need to accommodate two key issues that reflect societal shifts in how expertise is viewed [3]. First, that significant uncertainty exists about the benefit versus harm ratio of many medical tests and treatments. Second, and largely because such uncertainty is increasingly being acknowledged, there is widening agreement that the unilateral imposition of professional opinion about how to manage clinical problems--an approach often labelled as paternalism--is no longer a valid mode of interaction in healthcare settings. We recognise that patients have always acted as autonomous agents when it comes to their actual behaviour, a phenomenon that largely explains low adherence to prescribed medication. As a consequence, interactions in healthcare now need to acknowledge that often a balance exists between harm and benefit of different options--a concept we call 'equipoise' and that we will describe in greater detail [4]. In short, patients are increasingly expecting to be informed and involved in the process of care. This shift towards collaboration is not only relevant when people face difficult decisions where there are high stakes, 'narrow window of opportunities,' and where outcomes are uncertain, but also in situations where people need to manage long-term conditions or might want to consider making changes to their lifestyles in order to reduce future risks--in other words, conditions where there are long timeframes and, although the stakes are still high, the urgency is less pressing.

Health professionals are finding this role-shift challenging, and although pre- and postgraduate training curricula have adopted patient-centred models over the last two decades, there is evidence that practitioners struggle to incorporate patient agendas [5], seldom ask about their fears and expectations, and are unable to put shared decision making and behaviour change counselling into practice into their day-to-day routines. Although we could conjecture about the underpinning reasons for this, we focus this article on the interest that has emerged over the last decade in interventions to more actively involve patients in their care, be they in paper-based formats such as leaflets or booklets, or videotapes, compact video disks, or web-based tools. We wish to place these tools in the context of the wider literature concerned with the development and implementation of complex interventions to implement 'best practice' [6]. In addition, we wish to define the core components of these interventions in order to clarify the minimal requirements for classification, and to examine whether or not there is a need to have more than one class of intervention support.

## Discussion

In the late 1980s, a group of clinician-researchers in Boston [7] built on Wennberg's research showing that medical practice variation cannot be explained by varying disease prevalence [8]. Wennberg proposed dividing medical care into different types of care, which include 'effective' and 'preference-sensitive' care. Effective care is founded on strong evidence of effectiveness, which patients should always receive, where indicated. Preference-sensitive care on the other hand describes a situation where the evidence for the superiority of one treatment over another is not available; there are therefore two or more valid approaches to care and the best choice depends on how a patient values the risks and benefits of the treatments available. This work led to the definitions of unwarranted variation in preference-sensitive care as being due to a combination of different professional practice typically seen in different geographical areas, and a failure to adequately incorporate patient preferences into decision-making processes [9]. This distinction is ratified by the Grading of Recommendations Assessment, Development and Evaluation (GRADE) Working Group, which draws distinction between strong and weak practice recommendations, based on the quality rating of evidence, and also advocates that patient preferences are key to decision making in situations where weak recommendations exist [10].

Therefore, the need for clinicians to work collaboratively with patients in preference-sensitive decisions led to the creation of shared decision-making programs and the establishment, over time, of the Foundation for Informed Medical Decision Making [11]. Around the same time, O'Connor published work on the elicitation of patient preferences [12]. In 1998, an article appeared in which the term 'decision aids' was used to describe an intervention designed to help women consider whether or not to use hormone replacement therapy [13]. This was to be the first of many descriptions and evaluations of decision aids at the Ottawa Health Research Institute, and was the basis for many developments in this field. It was from these beginnings that the work began of collating an evidence base for the effectiveness of decision aids, resulting in a Cochrane Review [14]. In summary, these interventions are reported to have positive outcomes, such as improved user knowledge, accuracy of risk perceptions, satisfaction with decision making, to patients taking more conservative approaches to healthcare, and to greater clarity about their personal preferences [14].

The most recent development has been the creation of a set of quality standards based on reviews of evidence pertaining to a number of relevant quality dimensions for these interventions. The work has been co-ordinated by the International Patient Decision Aids Collaboration (IPDAS). A two-round modified Delphi consensus process resulted in the publication of a checklist [1] and, more recently, the establishment of an instrument that is capable of generating a quality score that will facilitate a quality assessment service and benchmarking exercise [15]. There will, no doubt, be debate about the applicability of these standards and concerns about the tendency for standards to restrict innovation and experimentation. Nevertheless, they signal a need for developers and researchers to pay attention to the active ingredients of decision support interventions. In parallel, there are also indications that researchers who have been concerned with the development and quality of clinical guidelines are also working on the need to involve patients, not only to make them accessible and relevant to patients' needs, but also to involve patients in their production and evaluation [16]. It is a short step, therefore, before clinical guidelines, if they come to be explicit about the availability of treatment options in many situations, will also qualify to be considered as decision support interventions [17].

Significant investments are being made in the development of decision support interventions, more recently including tools to help patients deal with long-term conditions (chronic diseases) [18,19]. Considerable efforts are being undertaken to implement these tools in real-world settings, with many developers viewing these interventions as products in a marketplace, encouraged by policies that encourage the convergence of commoditised healthcare, informed choice, and client-centred service design [20,21]. However, many questions remain unresolved. The role of patients' stories (narratives or testimonials) in these interventions is debated [22]. Such narrative elements have undoubted impact, so how to achieve balance remains problematic. In addition, how to best support a deliberation process is unresolved. Should people be encouraged to undertake exercises to clarify their preferences and consider competing attributes of options, or should these processes be left implicit, leaving individuals to rely on inherent heuristic approaches [23] and intuition [24]. These uncertainties will remain until further research emerges, but these tools will nevertheless continue to be produced and promoted [20].

### Preference sensitive decisions: situations of dual equipoise

O'Connor's defines decision aids as 'interventions designed to help people make specific and deliberative choices among options by providing information about the options and outcomes that are relevant to a person's health status' [14]. This definition rests on the assumption that healthcare contexts exist where it is reasonable to offer choice [19]. We contend that this, in turn, rests on the concept of equipoise--the existence of options that are in balance in terms of their attractiveness, or that the outcomes are to, a degree at least, equally desirable (or possibly, undesirable) [4]. This balance between options need not be perfect, indeed it is doubtful whether for any one individual that perfect equipoise between choices ever exists; but insofar as is reasonable, equipoise can be deemed to exist when a majority of people would agree that it is reasonable to consider making a choice between competing options.

Most decision support interventions have been developed to tackle preference-sensitive decisions where equipoise exists. A good example is the situation where a woman has been diagnosed with early stage breast cancer and needs to decide whether to have surgery that removes or conserves the breast (mastectomy or breast conservation) [25]. In this situation, the decision is relatively urgent, cannot be deferred indefinitely, and, moreover, is a difficult one to make because there is more than one option that can be considered. Research indicates that the outcomes of mastectomy and breast conservation surgery are more or less comparable in terms of mortality, but that important differences exist for patients' quality of life [26,27]. Health professionals recognise this decision as one where there mortality outcomes are sufficiently equivalent for many individual circumstances to allow patient preferences to choose the surgical procedure. Patients, once knowing this equivalence, understand that it is a preference-sensitive decision because they place differential emphases on issues such as breast conservation, body image, sexuality, and recurrence rates of local cancer. This decision can therefore be considered to have dual equipoise, where both health professionals and patients, once informed, agree conceptually that individual preferences are acceptable arbitrators of choice. We propose that professionally-situated equipoise is a pre-condition to the existence of dual equipoise interactions, and that these in turn facilitate shared decision making, and, as a result, are a pre-condition for the implementation of decision support interventions. Examining this proposal, we suggest that dual equipoise helps both the professional and the patient accept the validity of discussing options, helps patients to understand why their preferences are relevant and that the option attributes deserve deliberative thought in which counterfactual (what if) situations are considered. In short, these interventions provide individuals with the opportunity to construct and forecast preferences about their short, intermediate, and long-term outcomes. Given these characteristics, the consideration of surgical options in early breast cancer unequivocally meets dual equipoise criteria--it has reasonable, available options that need to be carefully deliberated.

Yet, we need to immediately recognise that the acceptance of equipoise remains one of the most difficult issues in clinical practice and, in addition, for patients to understand, given that it requires an acceptance that there is no right answer. For example, the decision for a man to be tested for the prostate specific antigen is accepted to be preference-sensitive and where decision support interventions are advocated [28-30]. But in practice, neither patients nor medical practitioners act accordingly: the public perception, abetted by media campaigns, seldom, if ever promotes the concept of equipoise around this decision; so the role of decision support interventions, their provenance, and their promotion becomes vital, and even more controversial in determining the best course of action. There is a requirement to see decisions like this in a broader context and to consider consequences across longer-term time horizons.

These arguments bring us to a proposed definition: decision support interventions help people think about choices they face; they describe where and why choice exists, in short, conditions of dual equipoise; they provide information about options, including, where reasonable, the option of taking no action. These interventions help people to deliberate, independently or in collaboration with others, about options by considering relevant attributes; they support people to forecast how they might feel about short, intermediate, and long-term outcomes that have relevant consequences in ways that help the process of constructing preferences and eventual decision making appropriate to their individual situation [31].

This is a broad definition, but it does require clarity about the nature of equipoise and whether the equipoise is located in one or more actor in any given interaction, an issue to which we return. It requires information provision, and the presence of two or more options, accepting that taking no action is in some cases an acceptable option. Decision support interventions offer no guidance about exactly how individuals should undertake the task of deliberation. We take the view that we do not yet have sufficient evidence on which to stipulate such an addition. When we examine decision support interventions from this vantage point, we notice that the majority of interventions have tackled decisions that we regard as having dual equipoise, where professionals (or at least professionals who are willing to acknowledge uncertainty, in its many guises, and help patients become involved in decisions) are willing to spend time introducing the concept of choice and undertake the inevitable additional work of addressing the questions and anxieties that arise [32]. We need here to also address the issue of terminology. We have chosen to use the term 'decision support intervention' in preference to the more widely used term 'decision aid'. We do this in order to draw attention to the issue that the term aid may not sufficiently encompass the range of potential interventions that are being developed and tested, for example, the arrival of multi-media web-based interactive and collaborative social network media. We also will inevitably need to refocus our evaluation beyond the artefact itself and to recognise that these tools are examples of embedded complex interventions [6], where the issues of how they are used, when, and by who will contribute to as much to their potential impact as the content of the artefact.

### Situations without dual equipoise

It follows therefore that here are also situations that lack dual equipoise. These are situations where strong evidence exists in favour of specific treatments or tests, or where there is a clear consensus that one approach is superior over another or that a change in lifestyle leads to greater benefit than harm. In Wennberg's categorization, this is known as effective care. Perhaps some will argue that we are overlooking patients' rights of self-determination, and that the principles of patient autonomy should apply even when professionals hold views about effective care. This is not the case. Indeed, we argue that excellent clinicians will explore patient agendas to the full, no matter how much those agendas run counter to prevailing scientific views. However, we also wish to see patient involvement flourish in real clinical settings. In situations where benefits clearly outweigh harms, professionals will not regard them as having dual equipoise and the deliberations will not be considered worth the investment required to achieve shared decision making. In other words, we are pragmatists more than we are ethicists who support mandatory autonomy [33].

A good example of this kind of situation--and one that clinicians face daily--is supporting a patient managing a long-term condition. For the majority of these conditions, there is good evidence that links specific processes to good outcomes--either adhering to medication or modifying lifestyle. Achieving the goals of good control for high blood pressure, diabetes, managing kidney or heart failure requires continued engagement in a set of behaviours. Managing a chronic disease is therefore all about changing behaviour and sustaining new habits, not about making a decision at one point in time. Professionals are not in equipoise. Clinical practice guidelines clearly delineate how professionals should operate and what treatments and behaviour changes they should recommend. Patients however, often don't recognize that their current behaviours or lack of adoption of professionally recommended changes may be in conflict with their long-term goals of maximizing longevity or quality of life [34]. The professionals' task is to support the patient in understanding the situation, to set agendas, to address ambivalences, and ultimately to see the discrepancy between what they are doing and ultimately want for themselves. The role of approaches such as behaviour support interventions, such as motivational interviewing and behaviour change counselling, is clear in these situations. Some of the tools developed to help patients in this area have also been called decision aids, but might need re-conceptualisation as a different class of intervention [18,19].

Examining one example in depth, we consider a common situation. An overweight 50 year-old lorry driver recently diagnosed with diabetes is struggling to control his blood pressure and weight. The patient also faces the challenge of trying to give up smoking whilst also learning about diabetes and balancing his own preferences and other demands on his time. The clinician is an excellent communicator and, where he feels able, shares decisions with patients. However, despite his respect for the patient's right to autonomy and self-determination, the clinician feels professional responsibility to explain risks and consequences. Whereas there are opportunities to pose valid choices (such as a range of smoking cessation methods, or whether to prioritise weight loss versus blood pressure control), the professional feels there is a larger overriding goal and does not perceive the situation to be one of clinical equipoise. His agenda is to modify the individuals' risk profile and, although he aims to do that with sensitivity and tact, he nevertheless has a clear agenda to motivate the patient to adopt a healthier lifestyle and to better manage a long-term condition.

The patient however is ambivalent about the problem. He is knows many other drivers who have come to little harm from smoking and, besides, a beer with his friends is a valued escape from a tedious routine. He appreciates his clinician's concern and does his best to adhere to an agreed new medication regime, but he is, at best, ambivalent about whether to attempt all the suggested changes. This is a stark example perhaps, but dual equipoise is clearly not present. For the professional, the evidence points in a clear direction, while the patient has other competing priorities and preferences. We contend that interventions designed to deal with these kinds of problems are probably best regarded as behavioural support interventions rather than decision support interventions. Motivational interviewing and behaviour change counselling are good examples of such approaches, and are interventions aimed at supporting individuals to recognise actions that are important to them and to gain confidence in being able to sustain the behaviours over time [34]. This argument, in turn, brings us to a potential definition: behavioural support interventions describe, justify, and recommend actions that, over time, lead to predictable outcomes over short, intermediate, and long-term timeframes, and that have relevant and important consequences for those who are considering behaviour change.

### Definitions of decision and behaviour support interventions

From these two descriptive accounts, we move to compare the two definitions and to discuss their implications. Table 1 provides a summary of their key characteristics. These descriptions are deliberately brief: they provide only an outline of what such an intervention could eventually contain. The point is to draw attention to the issue of dual equipoise as a design determinant. Dual equipoise assumes that all parties in the decision space agree that preferences are paramount--that there is sufficient equivalence among options to allow personal preference to hold sway. In addition, such decisions are discrete in that they occur at a single time points, are often irreversible, and commonly, relatively urgent. A decision to undergo a surgical procedure, to have a test, to enter a screening programme--all these are decisions where dual equipoise exists, albeit to varying degrees, depending on ambient professional or policy perspectives. There are many ways in which interventions can be designed to address this decision-making episode, ranging from a brief description or comparison of options to elaborate interactive multimedia website. It remains to be seen whether or not such interventions will conform to standards such as those set by IPDAS, or indeed whether the IPDAS collaboration is nimble enough to adapt to innovations over time. At the core of the definition however, which is the rationale for putting it forward, is the assumption of dual equipoise, and unless an intervention is clear about the nature of a potential dual equipoise and the provenance of the evidence on which it makes such a claim, we contend that it cannot be classed as a decision support intervention.

**Table 1:** Definitions and key characteristics of decision and behaviour support interventions

**Definitions**	**Decision support interventions**	**Behaviour support interventions**
	'Decision support interventions help people think about choices they face; they describe where and why choice exists, in short, conditions of dual equipoise; they provide information about options, including, where reasonable, the option of taking no action. These interventions help people to deliberate, independently or in collaboration with others, about options by considering relevant attributes; they support people to forecast how they might feel about short, intermediate, and long-term outcomes that have relevant consequences, in ways that help the process of constructing preferences and eventual decision making appropriate to their individual situation'	'behavioural support interventions describe, justify, and recommend actions that, over time, lead to predictable outcomes over short, intermediate, and long-term timeframes, and that have relevant and important consequences for those who are considering behaviour' change'.
**Key characteristics**	Describe a decision where there is dual equipoise.	Describe the consequences (risks) of different behaviours/actions.
	Options are clearly delineated.	Options, if present, are ranked.
	Option attributes are clearly delineated and compared.	Describe a range of safe (risk reducing) behaviours/range of consequences of unsafe (risk enhancing) behaviours.
	Intermediate and long-term outcomes described, using social, psychological, and biological consequences, and decision-making processes and interventions are provided at cross-road points.	Intervention involves interaction, data collection, and feedback over time to support behaviour modification.
	A recommendation (decision or action) is avoided.	A recommendation is generated, albeit negotiated.

In situations where dual equipoise does not exist, the weight of evidence (or consensus) is such that a professional, to maintain professional and societal integrity, is swayed to recommend an action or to motivate an action or a change of behaviour. Helping a patient to achieve good self-care in diabetes or in heart failure entails a series of behaviours where benefits far outweigh risks, and therefore, ultimately, the clinician is obliged to set out an agenda that may be at odds with patient preferences. Similarly, there are many ways in which interventions can be designed to address the task of providing support for a recommended action or behaviour. A time-honoured method is the establishment of a continuing relationship with a supportive, informed clinician, as is exemplified by a primary care model. More recently, programmes that support the development of self-care and self-management have been developed [35-38]. Future programmes will no doubt build on these interventions, by enhancing patient motivation, creating patients capable of co-producing healthcare, and engaging patients more closely in monitoring their illness and reacting to data feedback methods. At the core, however, is the assumption that there are a set of actions and behaviours that will enhance patient healthcare outcomes that are not episodic recurrent decisions, but rather are automatically integrated into daily routines.

### Decision and behaviour support interventions: implications

This article proposes new and separate definitions for interventions that propose to help people arrive at high-quality decisions and to initiate and maintain behaviours that lead to improved outcomes in healthcare contexts. We are aware of previous definitions and hope the arguments put forward here help to clarify the debates surrounding the scope of these methods and the terminology being used. We further felt it necessary to clarify why these two classes of interventions are different from each other in terms of dual versus single equipoise, with the hope that we will prevent researchers and developers lumping together approaches that need different theoretical foundations [39]. Developers need to design tools that are clear about the different goals, characteristics, and motivations of users and place more emphasis on theories that align with the different tasks of either undertaking deliberative choices or initiating and sustaining behaviour change [40]. The important point here is that the theories that can guide development of decision support interventions (*e.g*., expected utility theory) are different from those that should guide development of behaviour support interventions (*e.g*., theory of planned behaviour, trans-theoretical model).

Although we agree that patient-centeredness is wide enough to apply to all health care interactions the concept of shared decision making applies best to situations where dual equipoise exists; behaviour change methods on the other hand, such as motivational interviewing or behaviour change counselling, applies to situations where dual equipoise is unavailable. We avoid going into more depth about these kind of interventions: there is a vast literature, and given the interest in self-management approaches to chronic diseases, it is likely that behaviour support interventions and the potential to harness the power of feedback from monitoring techniques and personalised interactive tools (web and telephony) will continue to be an area for further development. The design of decision support interventions, as they engage with web 2.0 and wiki technologies is also at a stage of evolution, and although standards are emerging, they will require modification as innovations and further research is published.

## Summary

We believe that, over time, both decision and behaviour support interventions will become important components of healthcare pathways. However, as we hope to have demonstrated, they have divergent aims, different relationships to equipoise and, by definition, form two intervention classes: decision and behaviour support interventions. By being clear about definitions and overall goals, we hope that our ability to use appropriate theories to design and evaluate their impact will also improve.

## Competing interests

The authors declare that they have no competing interests.

## Authors' contributions

GE and DF initiated the discussion with SR; all authors contributed to the final manuscript.

## Authors' information

Glyn Elwyn leads a research group on shared decision making at Cardiff University (decision laboratory http://www.decisionlaboratory.com) and co-leads the International Patient Decision Aids Standards Collaboration (IPDAS); Dominick Frosch has extensive experience in designing, evaluating, and implementing decision support interventions in the USA; Stephen Rollnick is an international expert on motivational interviewing and behaviour change.

## Funding

No funding.
